# Implementing School‐Associated Vaccination Events in Colorado Public Schools: A Statewide Survey of Nurses

**DOI:** 10.1111/josh.70232

**Published:** 2026-09-15

**Authors:** Liliane Nienstedt, Ashley Gromis, Laura J. Faherty, Ariella R. Korn

**Affiliations:** ^1^ RAND Pittsburgh Pennsylvania USA; ^2^ Vanderbilt University Nashville Tennessee USA; ^3^ RAND Santa Monica California USA; ^4^ RAND Boston Massachusetts USA; ^5^ MaineHealth Portland Maine USA; ^6^ Tufts University School of Medicine Boston Massachusetts USA

**Keywords:** consolidated framework for implementation research, COVID‐19, implementation facilitators and barriers, school nurses, school‐based vaccination

## Abstract

**Background:**

Schools are important settings for delivering vaccines. It is unknown how facilitators of and barriers to implementing school‐associated vaccination events (SAVEs) vary by school‐ and community‐level characteristics.

**Methods:**

We fielded a web‐based survey of school nurses that worked in Colorado public schools in Fall 2024 to assess perceived implementation facilitators and barriers. We linked survey responses (*n* = 368) to publicly available data and conducted descriptive analyses.

**Results:**

Key facilitators identified by nurses that held SAVEs between 2018 and 2024 included parental awareness and trust, and external partnerships. Barriers identified by nurses who did not hold SAVEs included lack of guidance, staff time, and external partnerships. Across school characteristics, lack of guidance was ranked as one of the three most important barriers. Nurses in rural schools were more likely to rank vaccine politicization as a top barrier. Staff time was a top barrier for nurses in smaller schools, schools serving families with lower incomes, and high schools.

**Implications for School Health Policy, Practice, and Equity:**

School nurses need guidance and external partnerships to successfully implement SAVEs.

**Conclusions:**

Context‐specific strategies to improve SAVE implementation will be critical to allow schools to contribute to keeping students, staff, and communities safe from vaccine‐preventable diseases.

Schools are important settings for delivering vaccines to students, staff, and families to reduce access barriers in traditional healthcare settings—particularly among rural, lower‐income, and racially or ethnically minoritized communities that experience disparities in vaccination rates [1, 2, 3, 4, 5, 6]. School vaccination programs often involve time‐limited events (e.g., pop‐up clinics, mobile vans, health fairs) that focus on mass delivery of vaccines and are coordinated between schools and external partners such as a health system or public health department. These events have been used to deliver many types of vaccines, including routine childhood and adolescent immunizations [7], seasonal influenza vaccines [8], catch‐up adolescent immunizations (e.g., Hepatitis A and B) [9], and pandemic vaccinations (e.g., H1N1 and COVID‐19) [10]. While they do not routinely administer vaccines, school nurses typically help coordinate and publicize these events, which we call “school‐associated vaccination events” (SAVEs) [1, 11, 12, 13]. SAVEs can also be referred to as “school‐located” vaccination events; however, we use “school‐associated” to acknowledge that not all events occur on school grounds.

The success of SAVEs depends on how they are implemented [13, 14, 15, 16, 17]. Existing evidence highlights programmatic factors that may contribute to vaccination outcomes, including processes for obtaining parent consent [3, 12, 13, 18, 19, 20, 21], vaccine education and promotion activities [22, 23, 24], event hours and locations [12, 25], availability and participation of school nursing staff [10, 13, 19], considerations of vaccine inventory, delivery, and storage [11, 12, 13, 22], and funding [3, 11, 13]. The success of SAVEs may also be impacted by factors beyond the school context, including availability of primary care [26], alternative community vaccination sites [18, 26, 27, 28], and local political climate [29, 30, 31, 32].

While various facilitators of and barriers to holding SAVEs have been documented, they have not yet been organized according to well‐established implementation science frameworks [33, 34]. Organizing findings in this way emphasizes the theoretical basis for our analysis; helps structure the presentation of a large number of facilitators and barriers (hereafter “implementation determinants”); and helps researchers and practitioners better understand and tailor interventions to address these factors. Furthermore, a key knowledge gap is how these implementation determinants vary across schools and communities. This information could inform context‐specific strategies to improve the implementation of equity‐centered SAVEs [3, 14]. Finally, we lack a comprehensive understanding of public school nurses' perspectives on SAVE implementation. Nurses' insights may generate valuable guidance for SAVE implementation processes and schools considering holding a SAVE for the first time.

To address these gaps, this study (1) describes school nurses' perceptions of SAVE implementation determinants in Colorado public schools between 2018 and 2024 and (2) examines associations of implementation barriers with school and community characteristics.

## 1 Methods

### 1.1 Participants

Study participants were school nurses that worked in at least one Colorado public school at the time of survey administration in fall 2024. Participants were recruited through two listservs of school nurses maintained by the Colorado Department of Education (CDE) and the Colorado Association of School Nurses (CASN). There were 864 public school nurses working in 1597 public schools in Colorado in the 2023–2024 school year [35]. In Colorado, over 94% of children attend public school [36]. Thirty‐three percent of Colorado school‐aged children are Latino [37], nearly 35% of Coloradans are publicly insured [38], and more than half of Colorado counties are frontier or rural [39]—all historically underserved communities in traditional healthcare settings [40, 41]. In Colorado, there are also substantial gaps in the share of children receiving all recommended vaccinations by health insurance type. Over 80% of children with private insurance have received all recommended vaccines, compared to under 60% of children insured by Medicaid—the largest gap of any state [42]. Thus, Colorado is an important context for understanding equity considerations in SAVE implementation.

### 1.2 Procedure

Colorado public school nurses were invited to complete a self‐administered, web‐based survey that was estimated to take 15–20 min to complete. Our Colorado‐based partners posted the invitation and two additional reminders with the survey link on the CDE and CASN school nurse listservs. In the survey's introduction, nurses were instructed to gather records, logs, calendars, or other materials documenting SAVEs held from July 1, 2018, to June 30, 2024. This period was chosen to capture SAVE availability before, during, and after the COVID‐19 Public Health Emergency (PHE), when SAVEs may have been adapting to changing public health needs. Participation was voluntary, and nurses provided electronic consent. Participants received a $50 gift card upon survey completion.

### 1.3 Instrumentation

The study team developed the instrument based on a review of the school‐located vaccination literature, and in consultation with a school nurse partner in Colorado. Survey items assessing school nurses' perceptions of implementation determinants were informed by the Consolidated Framework for Implementation Research (CFIR), a widely used implementation science framework that conceptualizes links between implementation determinants and implementation effectiveness [43, 44, 45]. Key CFIR domains characterize the: *Innovation* (i.e., SAVEs); *Implementation Process* or strategies to carry out SAVEs; roles and characteristics of *Individuals* involved with implementing and participating in SAVEs (e.g., school nurses, school leaders, public health and healthcare partners, caregivers, students); *Inner Setting* in which implementation occurs (i.e., schools); and *Outer Setting*, representing the school district (hereafter, “district”) and broader community.

**FIGURE 1:**
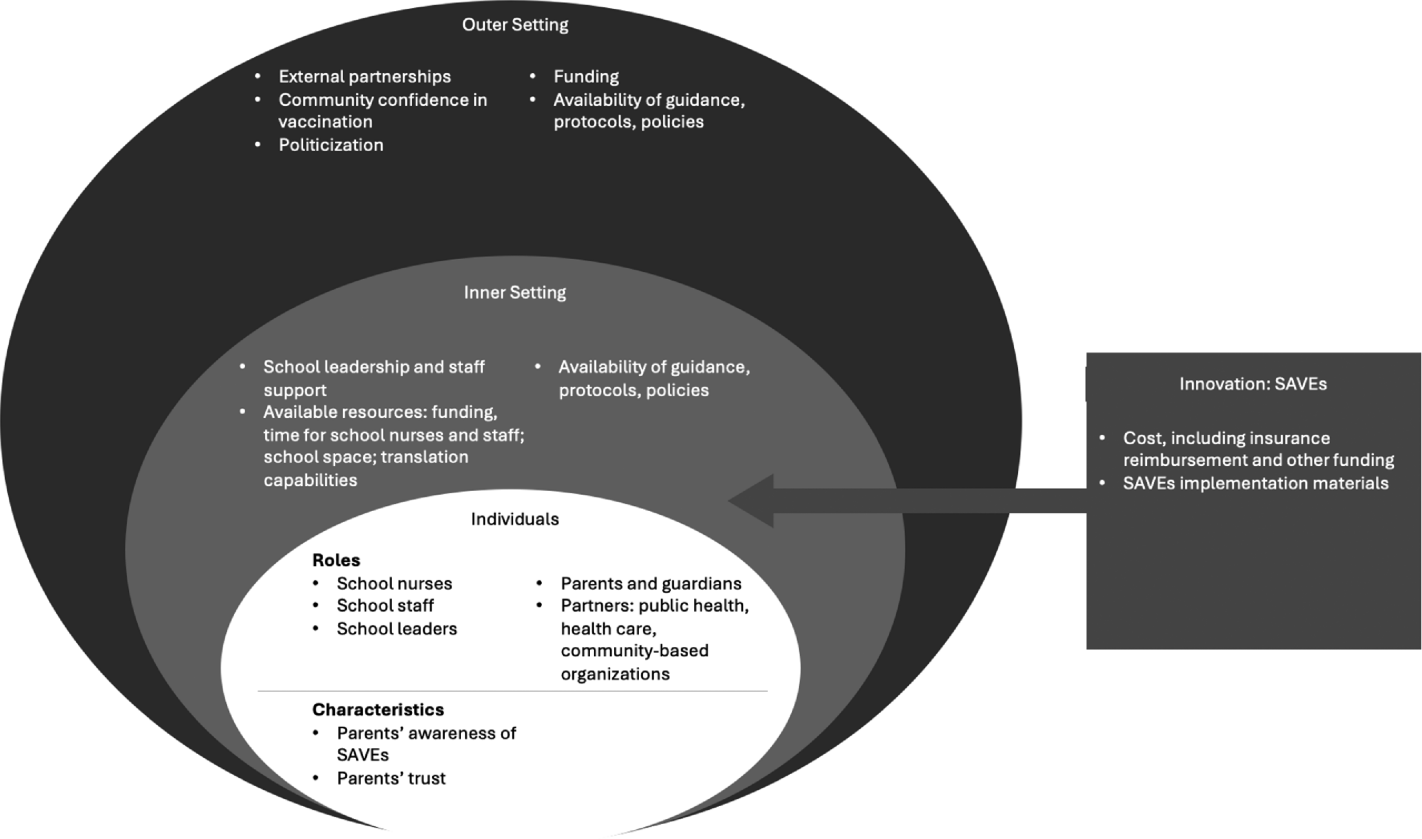
SAVEs implementation determinants included in survey instrument, organized by the Consolidated Framework for Implementation Research (CFIR) [44].

School nurses were asked to report the number of “temporary, time‐limited vaccination events” offered by their school(s) during the study period. If nurses worked in a school where no SAVEs were held, they were asked if SAVEs would have been helpful but were not held for one or more reasons to capture perceived SAVE need. Nurses at schools that held at least one SAVE during this period were asked to rate their extent of agreement on a 5‐point Likert scale ranging from “Strongly Agree” to “Strongly Disagree,” with 17 factors that may have helped or hindered SAVE implementation (Table 1A, in Supporting Information). Nurses at schools that did not hold SAVEs but reported that they would have been helpful rated the extent to which those same factors (except one factor that did not apply: parental awareness of SAVEs) were reasons that schools did not hold vaccination events. For each of the 16 potential barriers, nurses selected “not a reason,” “somewhat important reason,” or “very important reason” for not holding a SAVE. Nurses then ranked their top three barriers. The survey's skip logic is shown in Figure 1A in Supporting Information. Figure 1 shows the implementation determinants included in the survey, organized by CFIR domain. The survey also included open‐ended fields for additional reasons SAVEs were or were not held and general comments on school vaccination.

### 1.4 Data Analysis

The survey was completed by 368 nurses, representing approximately 43% of Colorado public school nurses in the 2023–2024 school year. Nurses reported the name(s) and address(es) of all the school(s) at which they currently worked, totaling 725 school locations (average: 2 per nurse; range: 1–5). We excluded 61 entities that did not meet the study inclusion criteria: schools that opened after the study period (*n* = 1); homeschool sites (*n* = 1); virtual schools (*n* = 12); private schools (*n* = 2); childcare centers (*n* = 17); district‐level responses (*n* = 13); and duplicate schools (*n* = 15).

Using nurse‐reported school names and addresses, we matched the remaining 664 schools (associated with 346 nurses) to CDE and National Center for Education Statistics (NCES) data to obtain school characteristics (CFIR Inner Setting). Due to minor differences between school information in the survey data and CDE/NCES data, we performed a series of probabilistic merges using the “reclink” package in Stata 17.0. We linked all records in which the school's name and address (street number, city, ZIP Code) were exact matches. We manually reviewed all remaining probabilistically matched records to verify the correct linkage. Of the 664 schools, we were able to match 627 (94.4%). We excluded 37 unmatched schools. Our final analytic sample included 330 nurses reporting on 627 schools (Figure 1A in Supporting Information). We obtained data on school enrollment, student demographics, location in the Denver Metro Area from CDE, and locale designation (e.g., urban, rural) and location (latitude/longitude) from NCES.

To approximate the local sociodemographic and health care context (CFIR Outer Setting), we linked schools to publicly available county‐level data. We obtained estimates for population, poverty rate, and median household income from the 2018 to 2022 5‐year American Community Survey. We obtained the number of primary care physicians from the 2023 to 2024 Department of Health and Human Services Area Health Resources File [46] and percentage uninsured population ages 19 years and under from the Small Area Health Insurance Estimates Program [47]. We calculated the rate of primary care physicians per 1000 people by dividing the number of primary care physicians by county population. We linked schools to the Health Resources and Services Administration's Medically Underserved Areas using the NCES school location [48].

We calculated descriptive statistics for closed‐ended survey items and school‐ and community‐level characteristics. T‐statistics were calculated from a two‐sample *t*‐test using Welch's approximation to allow for unequal variance in the comparison groups. Qualitative data from open‐ended survey items were analyzed using content analysis to identify implementation determinants not assessed in the survey (Table 1A in Supporting Information). We applied a dual, non‐independent approach to coding emergent themes: one researcher coded the open‐ended responses, and a second researcher reviewed the codes. Discrepancies were resolved through discussion and consultation with a third researcher.

## 2 Results

Compared to Colorado public schools with a nurse in the 2023–2024 school year (*n* = 1597), our sample (627 schools) was less likely to include charter schools and schools spanning multiple grade levels (“span schools”) and more likely to include elementary schools (Table 2A in Supporting Information). Sample schools had higher student enrollments (537.6 vs. 494.4) and larger shares of Hispanic/Latino (39.8% vs. 37.1%) and other race/ethnicity students (9.1% vs. 8.5%). Sample schools were more often located in urban areas and counties with lower shares of uninsured children. Sample schools were located across 36 (56.3%) Colorado counties.

Nurses at 159 schools in our sample (25.3%) reported holding at least one SAVE; however, nurses at 44 of those schools reported SAVEs outside the study period or with insufficient detail to determine an event date. These 44 schools were categorized as not holding a SAVE during the study period. Schools with SAVEs within the study period (*n* = 115) differed from schools without SAVEs (*n* = 512) in several ways: those with SAVEs were more likely to be high schools, had larger shares of Hispanic/Latino and free/reduced‐price lunch (FRPL)‐eligible students, were located in towns (vs. urban, suburban, or rural areas), and were more likely to be in counties with lower median household income and higher shares of uninsured children (Table 1).

**TABLE 1:** Colorado sample school and community characteristics, by whether SAVEs were held and nurses' perceived SAVE need, 2018–2024.

	Held at least one SAVE in study period?					Schools without SAVEs in study period (*n* = 512)^b^				
	Yes		No^a^			Would have been helpful		Not needed		
	115		512			257		126		
Schools (*N*)	Mean	(SD)	Mean	(SD)	t‐stat^c^	Mean	(SD)	Mean	(SD)	t‐stat^c^
School characteristics										
School enrollment, *n*	554.4	(431.7)	519.5	(417.2)	−0.79	477.6	(381.4)	606.0	(464.4)	**2.69**
Charter School, %	5.2	(22.3)	10.9	(31.2)	**2.29**	10.9	(31.2)	15.1	(35.9)	1.12
Elementary school, %	39.1	(49.0)	55.1	(49.8)	**3.14**	56.0	(49.7)	53.2	(50.1)	−0.53
Middle school, %	19.1	(39.5)	13.5	(34.2)	−1.42	15.6	(36.3)	9.5	(29.5)	−1.74
High school, %	24.3	(43.1)	15.4	(36.2)	**−2.06**	13.2	(33.9)	16.7	(37.4)	0.87
Span school, %	17.4	(38.1)	16.0	(36.7)	−0.35	15.2	(35.9)	20.6	(40.6)	1.28
Black/African American, %	3.7	(5.3)	3.8	(5.9)	0.16	3.7	(5.2)	2.9	(4.9)	−1.40
Hispanic/Latino, %	45.6	(24.5)	37.3	(23.9)	**−3.31**	36.7	(22.7)	31.1	(22.2)	**−2.28**
Non‐Hispanic White, %	43.0	(26.5)	49.6	(24.9)	**2.43**	50.9	(24.5)	54.8	(23.0)	1.52
Other race,^d^ %	7.6	(5.4)	9.3	(7.5)	**2.79**	8.7	(5.6)	11.2	(11.0)	**2.32**
Free/reduced‐price lunch, %	57.8	(25.3)	49.6	(26.1)	**−3.11**	50.8	(26.0)	40.3	(24.5)	**−3.80**
NCES locale designation										
Urban, %	37.4	(48.6)	43.8	(49.7)	1.26	44.4	(49.8)	39.7	(49.1)	−0.87
Suburban, %	15.7	(36.5)	32.4	(46.9)	**4.21**	28.8	(45.4)	38.1	(48.8)	1.79
Town, %	26.1	(44.1)	8.0	(27.2)	**−4.22**	7.8	(26.8)	7.9	(27.1)	0.05
Rural, %	20.9	(40.8)	15.8	(36.5)	−1.22	19.1	(39.4)	14.3	(35.1)	−1.20
In Denver Metro Area^e^, %	37.4	(48.6)	49.2	(50.0)	**2.35**	43.6	(49.7)	55.6	(49.9)	**2.21**
Community characteristics										
Median income (in $1000)	82.2	(14.4)	88.7	(18.9)	**4.10**	87.8	(19.4)	90.5	(20.1)	1.26
Families below poverty level, %	6.8	(2.7)	6.2	(2.7)	**−2.23**	6.4	(2.9)	5.9	(2.8)	−1.61
< 19 without health insurance, %	5.6	(1.2)	4.9	(1.3)	**−5.10**	4.8	(1.3)	4.7	(1.3)	−1.08
Physicians per 1000 population	0.8	(0.3)	0.8	(0.3)	0.75	0.8	(0.3)	0.8	(0.3)	0.11
Medically underserved Area, %	37.4	(48.6)	28.7	(45.3)	−1.75	27.2	(44.6)	25.4	(43.7)	−0.38

^a^ The 44 schools with SAVEs only reported outside the study period or without clear event dates are excluded.

^b^ The 44 schools with SAVEs only reported outside the study period or without clear event dates were missing data for this question. For an additional 85 schools, nurses said there was another reason why SAVEs were not held in the study period.

^c^ T‐statistics calculated from a two‐sample *t*‐test using Welch's approximation. Bold indicates *p* < 0.05.

^d^ Other includes Asian, American Indian and Alaska Native, Native Hawaiian and Other Pacific Islander, and multi‐racial students.

^e^ The Denver Metro area categorization is not mutually exclusive with the National Center for Education Statistics (NCES) locale.

Of the 512 schools with no reported SAVEs in the study period, nurses serving 257 of these schools (50.2%) said that SAVEs would have been helpful (Figure 1A in Supporting Information). Compared with schools where nurses did not think a SAVE was needed, these 257 schools had smaller enrollments, higher shares of Hispanic/Latino and FRPL‐eligible students, lower shares of students of other races/ethnicities, and were less likely to be in the Denver metro area (Table 1).

### 2.1 Factors Influencing Nurses' Ability to Hold SAVEs

Eighty‐three nurses worked in the 115 schools that held at least one SAVE during the study period. These nurses rated the extent to which a list of factors influenced their ability to hold SAVEs. The relevant CFIR domain(s) that correspond to each factor are noted in parentheses.

**FIGURE 2:**
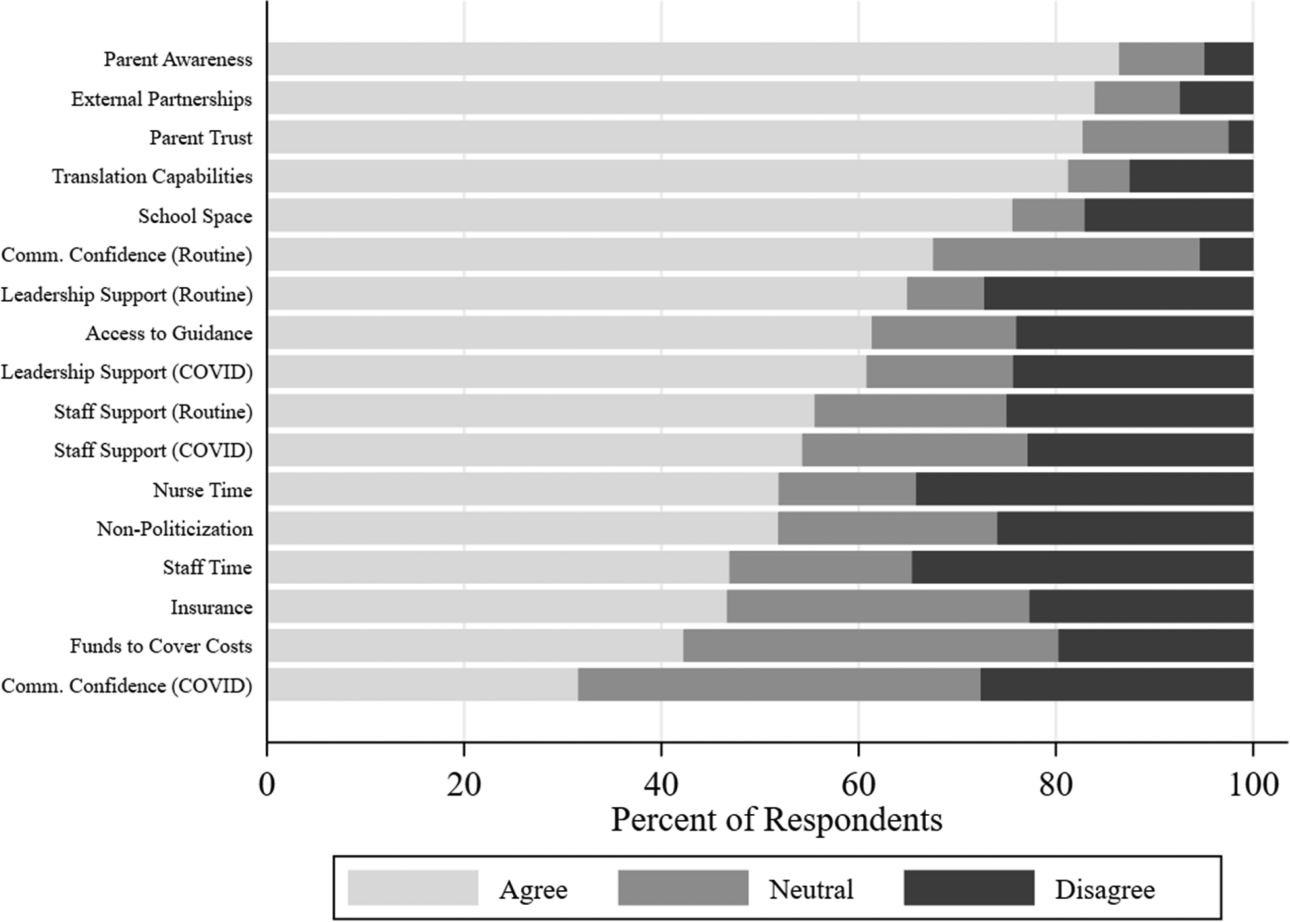
Factors influencing nurses' ability to hold SAVEs (*n* = 83). Nurses rated the extent to which these factors influenced their ability to hold School‐Associated Vaccination Events (SAVEs) using a 5‐point Likert scale. In this figure, “strongly agree” and “agree” were combined into “agree,” reflecting nurse agreement that these factors were influential as potential facilitators to implementing SAVEs. “Strongly disagree” and “disagree” were combined into “disagree,” signifying that nurses did not agree that these factors facilitated SAVE implementation. “Not applicable” and missing responses were excluded. The full set of responses is shown in Table 3A of the Supporting Information.

Nurses were most likely to agree or strongly agree that the following factors influenced their ability to implement SAVEs, reflecting potential facilitators: parental awareness of SAVEs (Individuals); partnerships with external organizations needed to conduct SAVEs (Outer Setting); parents trusting the school as a site to hold vaccination events (Individuals); and language translation capabilities (Inner Setting) (Figure 2). In contrast, nurses were most likely to disagree or strongly disagree that the following factors influenced their ability to hold their SAVEs, reflecting potential barriers: sufficient time for school staff to devote to vaccination (Individuals, Inner Setting); sufficient nurse time (Individuals, Inner Setting); community confidence in COVID vaccinations (Outer Setting); and leadership support to offer routine vaccines in schools (Individuals, Inner Setting). Table 3A of the in Supporting Information displays the full distribution of Likert scale responses and paraphrased illustrative quotations (to protect anonymity) about selected factors that supported SAVE implementation.

These nurses could also report other factors that “helped or hindered [your] ability to offer vaccines through your school(s).” Most nurses elaborated on implementation determinants already included in the survey (*n* = 38) or responded “not applicable” or “none” (*n* = 31); however, 14 nurses identified a new facilitator (e.g., timing SAVEs) to coincide with another school event, calling parents ahead of time whose children needed vaccines, and advertising the vaccines as free (all related to the Implementation process); a new barrier (*n* = 11) (e.g., low turnout, Individuals), lack of interoperability between the state's Immunization Information System and the school's documentation system (Implementation Process, Outer Setting), and a district Board of Education policy prohibiting vaccination in schools (Outer Setting); or a mix of both (*n* = 2).

### 2.2 Barriers Impeding Nurses' Ability to Hold SAVEs

**FIGURE 3:**
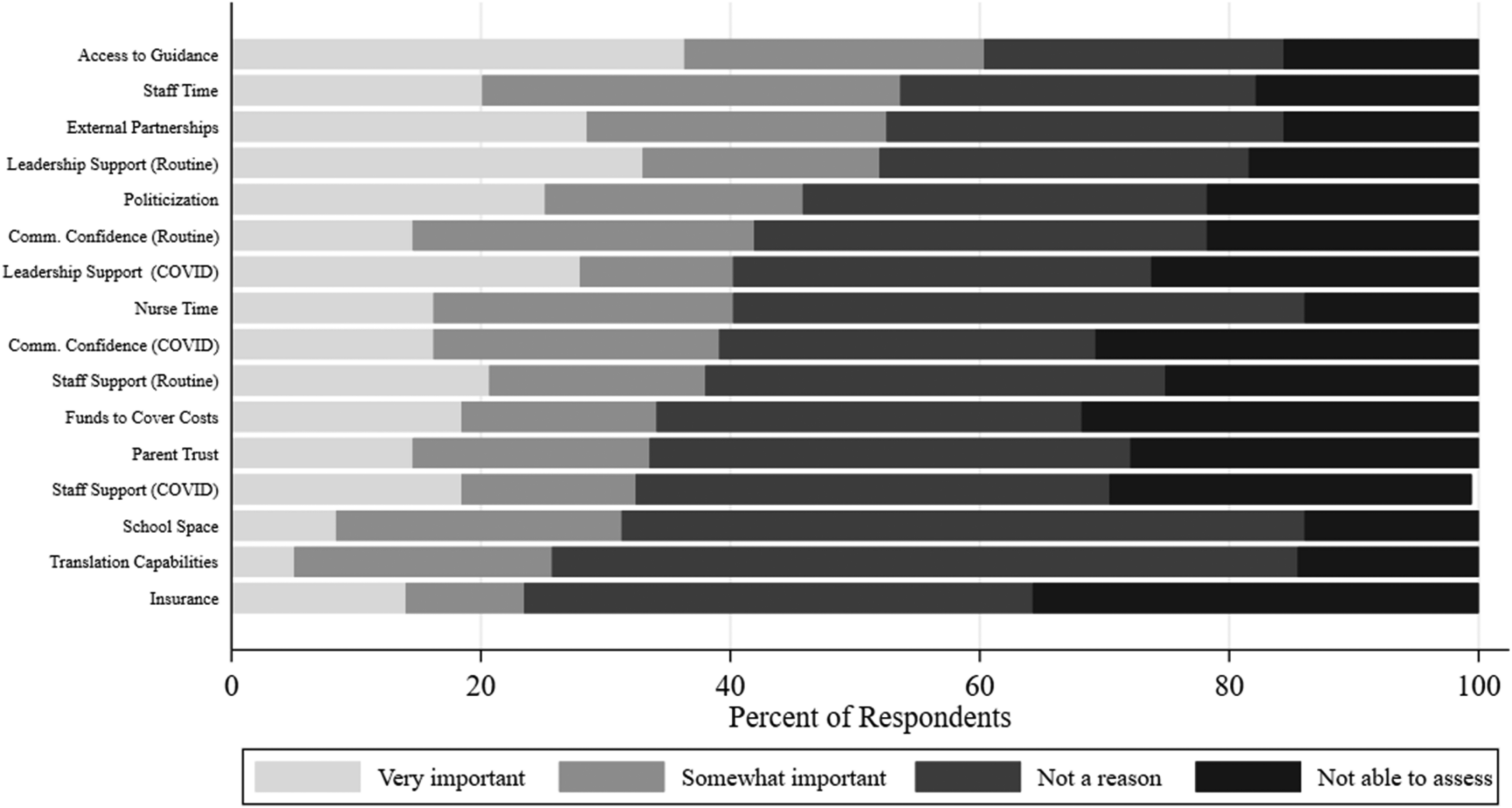
Potential reasons that schools did not hold SAVEs (*n* = 178). Frequencies of responses are shown in Table 4A of the Supporting Information. There were 179 nurses at schools without SAVEs in the study period; however, one nurse had missing data for these survey questions, resulting in 178 responses.

There were 179 nurses who worked at schools that did not hold a SAVE during the study period but reported that a SAVE would have been helpful or that there was another reason why a SAVE was not held. The most common barriers rated as “somewhat important” or “very important” reasons for not implementing SAVEs by these nurses included lack of guidance or policies on conducting SAVEs (Innovation, Inner Setting, Outer Setting); staff time constraints (Individuals, Inner Setting); lack of external partnerships (Outer Setting); and lack of leadership support to offer routine vaccines through the schools (Individuals, Inner Setting) (Figure 3). Nurses were least likely to report anticipated challenges with insurance reimbursement as an important barrier. Table 4A in Supporting Information includes the full range of survey responses and illustrative paraphrased quotations.

These 179 nurses were asked an open‐ended question about other factors that explained why SAVEs were not offered at their schools. Of the 178 who responded, 84 nurses said “none” or “not applicable,” 66 nurses elaborated on barriers included as survey items, and 21 nurses identified additional barriers not included in the survey. The remaining seven nurses reported sufficient vaccine access or high vaccination compliance without SAVEs, which we considered to represent lack of SAVE need (these schools may have similar characteristics to those without reported SAVE need shown in Table 1).

New barriers raised by more than one nurse included: transportation issues (Outer Setting); inconsistent district compliance with state vaccination policies (Outer Setting); coordination issues within and across districts and partners (Outer Setting); staff turnover (Inner Setting); nurses perceiving SAVEs as unnecessary due to historical low turnout (Individuals); and difficulty obtaining parental consent (Implementation Process). Two other barriers were raised by one nurse: lost instructional time (Inner Setting), and difficulty knowing which students qualify for the VFC program (Implementation Process).

Nurses who worked at schools without SAVEs were also asked to rank the top three barriers to holding SAVEs. Aligning with findings presented in Figure 3, the most frequently selected top barrier was lack of access to guidance on holding vaccination events (43.8%), followed by lack of external partnerships (31.5%), and staff time constraints (30.3%). Table 2 shows how top‐ranked barriers varied by school characteristics, including rurality, small school size, higher shares of FRPL‐eligible students, and grade level, with darker cells indicating barriers selected by a larger proportion of nurses. Across school characteristics, lack of access to guidance was a top barrier. Compared to the overall sample, nurses from rural schools were more likely to indicate that politicization was a top barrier and less likely to indicate that staff time was in their top three barriers. Nurses working in smaller schools and high schools also more frequently ranked constraints on nurses' time as a top barrier. While leadership support to offer routine vaccines was a common barrier across school characteristics, no nurses from small schools included it as a top barrier.

**TABLE 2:** Implementation factors' inclusion in top three barriers to holding SAVEs reported by nurses (%), by school and community characteristics (*n* = 178 responses).

		Nurses who worked at						
	All nurses	At least one rural school^a^	Schools with average enrollment <,200^b^	Schools with average > 75% FRPL^b^	At least one ES^a^	At least one MS^a^	At least one HS^a^	At least one Span School^a^
*N*	178^c^	38	12	46	122	53	57	48
Access to guidance	43.8	44.7	41.7	39.1	43.4	39.6	47.4	39.6
External partnerships	31.5	26.3	25	37	28.7	34	28.1	41.7
Staff time	30.3	18.4	41.7	41.3	27.9	34	40.4	16.7
Leadership support (routine)	27.5	31.6	0.0	28.3	30.3	32.1	24.6	27.1
Nurse time	25.8	23.7	58.3	32.6	23.8	24.5	38.6	20.8
Funding to cover costs	22.5	21.1	8.3	17.4	27	24.5	15.8	20.8
Politicization	20.8	34.2	8.3	2.2	20.5	20.8	19.3	20.8
Insurance	18.0	18.4	16.7	19.6	23	15.1	8.8	14.6
Comm confidence (routine)	14.0	21.1	16.7	17.4	13.9	13.2	17.5	10.4
Parent trust	11.0	15.8	16.7	4.3	13.1	13.2	8.8	14.6
Comm confidence (COVID)	9.0	15.8	16.7	10.9	6.6	9.4	12.3	10.4
Staff support (routine)	7.9	2.6	0.0	15.2	6.6	7.5	7	12.5
School space	6.7	5.3	16.7	10.9	5.7	9.4	8.8	4.2
Leadership support (COVID)	6.2	7.9	0.0	4.3	6.6	5.7	5.3	10.4
Translation capabilities	5.6	2.6	8.3	6.5	4.9	3.8	8.8	6.3
Staff support (COVID)	2.8	0.0	0.0	0.0	1.6	1.9	3.5	2.1

*Note:* Nurses were additionally able to select “other” and write in a response. These responses are excluded from the calculations in this table.

Abbreviations: ES, elementary school; FRPL, free/reduced‐price lunch; HS, high school; MS, middle school. Shading represents proportion of respondents who selected this answer, with darker cells indicating barriers selected by a larger proportion of nurses.

^a^ Nurses' responses were included in these columns if at least one of the schools in which they worked met the criteria (e.g., at least one school was located in a rural area, at least one school served elementary students).

^b^ Because nurses worked at multiple schools, the responses were grouped to nurses who worked at schools that, on average, had fewer than 200 students or served student populations where over 75% of students were eligible for FRPL.

^c^ There were 179 nurses at schools without SAVEs in the study period; however, one nurse had missing data for these survey questions, resulting in 178 responses.

### 2.3 Additional Implementation Factors and Synthesis by CFIR Domain

A total of 172 nurses responded to a final survey question in which they could provide any further comments about vaccination events. Notably, more than one‐third of responses (*n* = 61) reflected enthusiasm about previous or future SAVEs. New factors emerged in these responses, which we organize by CFIR domain below [44]. We present factors by Outer Setting, then Inner Setting, and finally Individual and Innovation Characteristics.

Multiple nurses noted that a barrier to SAVE implementation was that districts inconsistently enforce the state's school‐entry immunization policies (Outer Setting), influencing some districts' decisions not to hold SAVEs. For instance, one nurse remarked that their district did not hold a SAVE because it would have created “an appearance problem” if people perceived that their district was “forcing” vaccination as a requirement for attending school. Multiple nurses described district‐level hesitancy to hold SAVEs or enforce vaccination policy compliance because of fear of negative reactions from the community.

Nurses also perceived broader community sociodemographic factors and transportation access as barriers to holding SAVEs (Outer Setting). Multiple nurses shared that it was challenging to manage vaccination records for students who had newly arrived in the United States. One nurse commented that families in high‐poverty areas face multiple barriers to reaching clinics because they are “trying to juggle” many demands and prioritize “immediate needs.” Similarly, multiple nurses mentioned it was difficult for families to find transportation to SAVEs, which often required parents to be present to provide consent. Alternatively, some nurses at schools without SAVEs commented that a SAVE would have improved vaccination access for low‐income families by reducing transportation barriers to clinics.

Nurses also discussed small school size and staff turnover as factors that impacted their ability to hold SAVEs (Inner Setting). Nurses in smaller schools noted that they relied on district‐wide events or health department‐run mobile clinics. Additionally, many nurses were new in their roles, making it difficult to access information on how to hold a SAVE. As one new nurse put it, they were “unaware of the steps to take” to coordinate these events.

Reflecting the adaptability of SAVEs (Innovation), multiple nurses expressed interest in holding SAVEs via centrally‐organized mobile clinics to reduce burden on school staff. For example, one nurse commented that it would be helpful to have the district arrange mobile vaccination clinic visits on a rotating basis, visiting “certain schools [on] certain days” instead of having individual school nurses without “a lot of time and connection[s]” organize the mobile clinic's visit.

Finally, several nurses shared the importance of holding SAVEs at convenient times for parents (e.g., beginning or end of the school day, back‐to‐school events) to maximize their ability to “support their child and sign the [consent] paperwork” (Implementation Process).

## 3 Discussion

SAVEs may help close gaps in childhood vaccination rates, yet their implementation remains understudied. Using survey data from Colorado school nurses, our study identified factors across CFIR domains that influenced SAVEs implementation and differences across school and community characteristics. Nurses at schools that held SAVEs reported that implementation was most supported by parental awareness, external partnerships, and parent trust in schools as a location for vaccination. Key barriers identified by nurses who did not hold SAVEs included lack of guidance, staff time, and external partnerships. Nurses from rural schools were more likely to rank politicization of vaccines, a CFIR Outer Setting factor, as a top barrier to holding SAVEs. Staff time, an Inner Setting factor, was an important barrier for nurses working at smaller schools, schools serving families with lower incomes, and high schools.

Many nurses (60.3%) reported lack of guidance, protocols, or policies as a barrier to implementing SAVEs, which we categorized as part of the CFIR Innovation, Inner Setting, and/or Outer Setting to reflect variability of potential guidance source(s). The Association of Immunization Managers and National Association of School Nurses have previously highlighted available guidance from the National Association of County and City Health Officials [49] and the Association of State and Territorial Health Officials [50] for planning and holding school‐associated vaccination clinics; however, our findings suggest that many nurses may not be aware of these resources. In the open‐ended responses, multiple nurses shared that staff turnover made planning SAVEs difficult as well. Given high turnover and staffing shortages [51], improving nurses' access to SAVE guidance may enable schools to offer these events more consistently and be more efficient in their planning, especially when faced with staffing challenges.

As highlighted in prior work [14], nurses reported the importance of external partnerships for SAVE implementation, particularly with state and local health departments, emphasizing the importance of collaboration between education and health sectors (CFIR Outer Setting). Such partnerships may also help reduce burden among school staff and bolster parent trust. While not directly assessed in our survey, the COVID‐19 pandemic may have generated new external partnerships that improved schools' capacity to hold SAVEs post‐COVID. SAVE guidance should include resources and examples for building relationships with public health departments, health systems, local pharmacies, and community organizations.

Building on prior work related to COVID‐19 vaccine politicization [29, 30, 31, 32], we found that, overall, most nurses did not rank politicization as a major barrier; however, politicization was salient among nurses working in rural schools. Additionally, in open‐ended responses, multiple nurses shared that their districts did not strongly enforce state school vaccine requirements or avoided SAVEs due to political climates (CFIR Outer Setting). These findings highlight the complex, shifting political landscape that nurses must navigate in deciding whether to hold SAVEs.

Unlike some previous studies on school‐associated vaccination [3, 7, 11, 13, 52], few nurses cited funding as a SAVEs barrier. This finding may reflect that much of our study period coincided with the COVID‐19 PHE, when vaccines and their administration were largely federally funded, and states had flexibility in administering Medicaid and the Children's Health Insurance Program administration [53], alongside supplemental relief funds for states and schools [54].

## 4 Implications for School Health Policy, Practice, and Equity

Vaccines and vaccine recommendations are receiving renewed federal attention, yet school vaccine requirements are determined by state law and enforced locally by districts. This shifting national conversation may draw attention to how requirements are implemented and where vaccines are offered, particularly as childhood vaccination rates decline nationwide and vaccine‐preventable diseases become more common [55, 56, 57]. Our findings highlight the need for context‐specific strategies across CFIR domains to ensure schools remain accessible, trusted sites for preventive care for all children, especially those with less access to routine medical care. Policymakers at state and local departments of health and education can create resources and guidance for those interested in implementing SAVEs. Additionally, given that many nurses reported a perceived lack of guidance, additional support may be needed to ensure that school nurses, particularly those who are new to their roles, are aware of existing resources. Policymakers can also support collaborations between schools and community partners to help coordinate vaccination events. These strategies can be paired with continued efforts to strengthen parental awareness of, and trust in, opportunities for their children to receive vaccinations at school.

## 5 Limitations

First, our survey responses may reflect recency bias. While nurses were asked about SAVEs between 2018 and 2024, 43% began working at their current schools after 2022, likely emphasizing more recent experiences and leading to underreporting of SAVE frequency in earlier years. Second, at 44 schools, nurses erroneously provided SAVEs data outside the study period or with unclear event timing. We excluded these responses from analyses, preventing assessment of barriers for those schools. Third, while we received an estimated 43% response rate among licensed Colorado public school nurses, our sample was not representative of public schools with nurses (Table 2A in Supporting Information). Finally, due to likely underreporting of SAVE frequency and small subsample sizes when stratifying by school characteristics (e.g., rural schools, small schools), we could not robustly estimate relationships between school or community characteristics and SAVEs availability, nor analyze frequency of SAVEs implementation determinants by intersecting characteristics (e.g., small rural versus small urban schools).

## 6 Conclusion

This study offers new evidence on SAVEs implementation in Colorado between 2018 and 2024, providing among the first statewide data spanning the COVID‐19 pandemic and organizing implementation factors according to a well‐known implementation science framework. Strong external partnerships were consistently noted as a key determinant of SAVEs implementation. Unlike previous findings, financial barriers were rarely noted, while lack of guidance, limited staff time, and weak external partnerships emerged as key obstacles. Pandemic‐era funding and insurance flexibility likely supported SAVEs efforts and strengthened collaboration between health agencies and schools. However, post‐COVID, schools face declining childhood vaccination rates and increased vaccine‐preventable disease transmission. This research points to key factors associated with Individuals, the Innovation, Implementation Process, and Inner and Outer Settings that can be addressed to support SAVEs implementation in different contexts to improve equitable vaccination outcomes among school‐aged children.

## Funding

This work was supported by the National Institutes of Health (R01MD019798).

## Ethics Statement

This study was reviewed and approved by the RAND Human Subjects Protection Committee. Participants provided electronic informed consent prior to completing the survey.

## Conflicts of Interest

The authors declare no conflicts of interest.

## Supporting information


**Table 1A** Survey items assessing barriers and facilitators to implementing SAVEs.
**Figure 1A**. Flow diagram of school nurse survey nurses.
**Table 2A**. Characteristics of Colorado public schools with nurses compared to sample schools.
**Table 3A**. Factors influencing nurses' ability to hold SAVEs (*n* = 83).
**Table 4A**. Barriers influencing nurses' ability to hold SAVEs (*n* = 179).

## Data Availability

The data that support the findings of this study are available on request from the corresponding author. The data are not publicly available due to privacy or ethical restrictions.
