# Supplementary material for: Implementing School‐Associated Vaccination Events in Colorado Public Schools: A Statewide Survey of Nurses
Source: J Sch Health. 2026 Sep 15;96(10):e70232. doi: 10.1111/josh.70232 (PMC13575715; doi:10.1111/josh.70232)
Supplement: Supplementary file 1 [file JOSH-96-0-s001.docx]

**SUPPLEMENTARY MATERIAL**

**Table 1A.** Survey items assessing barriers and facilitators to implementing SAVEs

| Label | Factors that influenced nurses’ ability to provide SAVEs ^a^ | Reasons that SAVEs were not held^b^ | CFIR domain(s) |
| --- | --- | --- | --- |
| Nurse Time | I had enough time to devote to holding school-associated vaccination events | Constraints on my time | Individuals; Inner Setting |
| Staff Time | Other school staff had enough time to help with school-associated vaccination events | Constraints on the time of other school staff who would be needed to conduct a school-associated vaccination event | Individuals; Inner Setting |
| Parent Awareness | Parents were aware that the school was offering school-associated vaccination events | *Not assessed (not applicable for schools that did not offer SAVEs)* | Individuals |
| Parent Trust | Parents trusted the school as a site, or sponsor, of vaccination events | Parents’ lack of trust in vaccinations being offered through schools | Individuals |
| Staff Support (Routine) | I had support from other school staff to offer routine vaccines through the school | Lack of support from school staff to offer routine vaccines through the school | Individuals; Inner Setting |
| Staff Support (COVID) | I had support from other school staff to offer COVID-19 vaccines through the school | Lack of support from school staff to offer COVID-19 vaccines through the school | Individuals; Inner Setting |
| Leadership Support (Routine) | I had support from school leadership to offer routine vaccines through the school | Lack of support from school leadership to offer routine vaccines through the school | Individuals; Inner Setting |
| Leadership Support (COVID) | I had support from school leadership to offer COVID-19 vaccines through the school | Lack of support from school leadership to offer COVID-19 vaccines through the school | Individuals; Inner Setting |
| Translation Capabilities | Our school was able to communicate with families who speak a language other than English about vaccinations being offered through the school | Limited ability to communicate with families who speak a language other than English about vaccinations being offered through the school | Inner Setting |
| School Space | Our school had enough space to hold school-associated vaccination events | Lack of space for conducting school-associated vaccination events | Inner Setting |
| Access to Guidance | I had access to guidance, protocols, or policies on conducting school-associated vaccination events (e.g., consent processes) | Lack of guidance, protocols, or policies on conducting school-associated vaccination events | Inner Setting and/or Outer Setting; Innovation |
| Insurance | Our partners could cover their costs of conducting school-associated vaccination events through insurance reimbursements | Anticipated challenges with covering costs through insurance reimbursement | Outer Setting; Innovation |
| Funds to Cover Costs | Our school or partners received sufficient other funding, aside from insurance reimbursements, to cover the costs of holding school-associated vaccination events | Lack of other funding, aside from insurance reimbursement, to cover costs of holding school-associated vaccination events | Inner Setting and/or Outer Setting; Innovation |
| External Partnerships | Our school had partnerships with external organizations (e.g., local health department, health system, community-based organizations) needed to conduct school-associated vaccination events | Lack of partnerships with external organizations (e.g., local health department, health system, community-based organizations) needed to conduct school-associated vaccination events | Outer Setting |
| Comm Confidence (COVID) | People in the community surrounding the school (parents and other community members) had confidence in COVID-19 vaccination | Lack of confidence among people in the community surrounding the school (parents and other community members) in COVID-19 vaccination | Outer Setting |
| Comm Confidence (Routine) | People in the community surrounding the school (parents and other community members) had confidence in routine vaccination (e.g., measles, whooping cough, influenza) | Lack of confidence among people in the community surrounding the school (parents and other community members) in routine vaccination (e.g., measles, whooping cough, influenza) | Outer Setting |
| Politicization | In the community where the school is located, vaccinations or vaccine requirements being politicized hindered my school's ability to hold school-associated vaccination events^c^ | The politicization of vaccinations or vaccine requirements in the community where the school is located | Outer Setting |

^a^ Response options included: strongly disagree; disagree; neither disagree nor agree; agree; strongly agree; not applicable.

^b^ Response options included: this was not a reason my school(s) didn’t hold vaccination events; this was a somewhat important reason my school(s) didn’t hold vaccination events; this was a very important reason my school(s) didn’t hold vaccination events; unable to assess.

^c^ We reverse-coded this factor in our analysis of facilitators such that it represented politicization of vaccines or vaccine requirements not hindering a school’s ability to hold SAVEs.

**Figure 1A.** Flow diagram of school nurse survey nurses

Note: Only schools (not nurses) are mutually exclusive in this figure. Some nurses work at multiple schools, and in some cases, these schools varied in whether they held SAVEs or not, and if not, whether SAVEs would have been helpful or if there was another reason for not holding them. Some nurses responded to both the “implementation barriers” and “implementation factors” questions.

**Table 2A.** Characteristics of Colorado public schools with nurses compared to sample schools

|  | CO Public schools with licensed nurses | | Schools in sample^a^ | | Schools not in sample | |  |
| --- | --- | --- | --- | --- | --- | --- | --- |
| Number of schools | 1,597 | | 585 | | 1,012 | |  |
|  | Mean (SD) | | Mean (SD) | | Mean (SD) | | t-stat^b^ |
| **School Characteristics** |  | |  | |  | |  |
| *Demographics* |  | |  | |  | |  |
| School Enrollment, *n* | 494.4 | (408.7) | 537.6 | (424.6) | 469.5 | (397.3) | **3.16** |
| Charter School, % | 13.8 | (34.5) | 9.9 | (29.9) | 16.1 | (36.8) | **-3.66** |
| Elementary School, % | 48.5 | (50.0) | 51.8 | (50.0) | 46.6 | (49.9) | **2.00** |
| Middle School, % | 15.8 | (36.5) | 14.2 | (34.9) | 16.7 | (37.3) | -1.34 |
| High School, % | 17 | (37.6) | 17.9 | (38.4) | 16.5 | (37.1) | 0.71 |
| Span School, % | 18.7 | (39.0) | 16.1 | (36.8) | 20.2 | (40.1) | **-2.08** |
| *Student Demographics* |  | |  | |  | |  |
| Black/African American, % | 4.3 | (7.2) | 3.9 | (6.0) | 4.5 | (7.8) | -1.72 |
| Hispanic/Latino, % | 37.1 | (24.7) | 39.8 | (24.4) | 35.5 | (24.7) | **3.38** |
| non-Hispanic White, % | 50.2 | (26.3) | 47.2 | (25.3) | 51.9 | (26.7) | **-3.50** |
| Other race^c^, % | 8.5 | (6.5) | 9.1 | (7.3) | 8.1 | (6.0) | **2.81** |
| Free/Reduced-Price Lunch, % | 49.9 | (25.7) | 51.6 | (26.4) | 49 | (25.3) | 1.93 |
| *Location* |  | |  | |  | |  |
| NCES Locale Designation |  |  |  |  |  |  |  |
| Urban, % | 38.3 | (48.6) | 43.6 | (49.6) | 35.2 | (47.8) | **3.30** |
| Suburban, % | 29 | (45.4) | 29.2 | (45.5) | 28.9 | (45.3) | 0.13 |
| Town, % | 11.6 | (32.0) | 11.3 | (31.7) | 11.8 | (32.2) | -0.30 |
| Rural, % | 21.2 | (40.9) | 15.9 | (36.6) | 24.2 | (42.9) | **-4.09** |
| In Denver Metro area^d^, % | 47.6 | (50.0) | 48.2 | (50.0) | 47.2 | (49.9) | 0.39 |
| **Community Characteristics** |  | |  | |  | |  |
| Median family income, (in $1000) | 86.7 | (18.8) | 87.8 | (18.2) | 86.1 | (19.1) | **1.77** |
| Families Below Poverty Level, % | 6.4 | (3.0) | 6.3 | (2.7) | 6.5 | (3.1) | -1.35 |
| <19 without health insurance, % | 5.2 | (1.4) | 5.1 | (1.3) | 5.3 | (1.4) | **-2.88** |
| Physicians per 1,000 population | 0.8 | (0.3) | 0.8 | (0.3) | 0.8 | (0.3) | 0.00 |
| Medically Underserved Area, % | 30.8 | (46.2) | 28.4 | (45.1) | 32.2 | (46.8) | -1.60 |

^a^ Of the 627 schools in our sample, 39 (6%) were indicated as not having a licensed nurse available during school hours and 3 (< 1%) were missing these data. These 42 schools are excluded from this table.

^b^ T-statistics calculated from two-sample t-test using Welch’s approximation. Bold indicates t>1.96.

^c^ Other race includes Asian; American Indian and Alaska Native; Native Hawaiian and Other Pacific Islander; and multi-racial students.

^d^ The Denver Metro area categorization is not mutually exclusive with National Center for Education Statistics (NCES) locale.

**Table 3A.** Factors influencing nurses’ ability to hold SAVEs (n=83)

| **Factor**^a^ | **Strongly disagree** | **Disagree** | **Neutral** | **Agree** | **Strongly agree** | **N/A** | **Select illustrative context for factors nurses agreed facilitated their ability to hold SAVEs, paraphrased from free-text responses** |
| --- | --- | --- | --- | --- | --- | --- | --- |
|  | **n (%)** | | | | | |  |
| External Partnerships | 1 (1.2) | 5 (6.0) | 7 (8.4) | 30 (36.1) | 38 (45.8) | 2 (2.4) | - “Solid partnerships” with state and local health departments and local pharmacies have helped nurses “immeasurably.” These partnerships were established during COVID and have continued. - In one district, administrative staff from the district coordinated the mobile vaccination clinic, rather than the school nurses. |
| School Space | 4 (4.8) | 10 (12.1) | 6 (7.2) | 35 (42.2) | 27 (32.5) | 1 (1.2) |  |
| Translation Capabilities | 2 (2.4) | 8 (9.6) | 5 (6.0) | 39 (47.0) | 26 (31.3) | 3 (3.6) | - The health agency had nurses who spoke Spanish, which was critical as almost all student receiving vaccines spoke Spanish. The agency also streamlined the process of coordinating the SAVE as they reached out to the school nurse, provided all the necessary paperwork in both English and Spanish, and helped verify students’ insurance. - Bilingual staff at both the school and the county department of health helped make sure parents were aware of SAVEs and helped on the day of the event. |
| Parent Awareness | 3 (3.6) | 1 (1.2) | 7 (8.4) | 46 (55.4) | 24 (28.9) | 2 (2.4) | - Nurses commented that they reach out to families whose students need school-required vaccines. |
| Leadership Support (COVID) | 5 (6.0) | 13 (15.7) | 11 (13.3) | 25 (30.1) | 20 (24.1) | 9 (10.8) |  |
| Parent Trust | 0 (0.0) | 2 (2.4) | 12 (14.5) | 47 (56.6) | 20 (24.1) | 2 (2.4) | - SAVEs are a “valuable resource for our families.” They help “eliminate barriers” stemming from transportation challenges and “limited knowledge of local resources.” - “The trust that families place in schools” increases the likelihood that parents will have their children vaccinated at in this setting. Another nurse commented that vaccinating in the school setting gives families “a sense of security.” |
| Leadership Support (Routine) | 7 (8.4) | 14 (16.9) | 6 (7.2) | 31 (37.4) | 19 (22.9) | 6 (7.2) |  |
| Staff Support (COVID) | 4 (4.8) | 12 (14.5) | 16 (19.3) | 23 (27.7) | 15 (18.1) | 13 (15.7) |  |
| Insurance | 6 (7.2) | 11 (13.3) | 23 (27.7) | 22 (26.5) | 13 (15.7) | 8 (9.6) |  |
| Funds to Cover Costs | 4 (4.8) | 10 (12.1) | 27 (32.5) | 17 (20.5) | 13 (15.7) | 12 (14.5) |  |
| Access to Guidance | 3 (3.6) | 15 (18.1) | 11 (13.3) | 33 (39.8) | 13 (15.7) | 8 (9.6) |  |
| Staff Support (Routine) | 6 (7.2) | 12 (14.5) | 14 (16.9) | 27 (32.5) | 13 (15.7) | 11 (13.3) |  |
| Nurse Time | 10 (12.1) | 17 (20.5) | 11 (13.3) | 30 (36.1) | 11 (13.3) | 4 (4.8) | - One nurse noted that having a full time (5 days per week versus only 1-2 days/week) nurse at the school makes it much more feasible to hold SAVEs. |
| Staff Time | 10 (12.1) | 18 (21.7) | 15 (18.1) | 30 (36.1) | 8 (9.6) | 2 (2.4) |  |
| Politicization (reversed)^b^ | 4 (4.8) | 17 (20.5) | 18 (21.7) | 36 (43.4) | 6 (7.2) | 2 (2.4) |  |
| Comm Confidence (COVID) | 4 (4.8) | 17 (20.5) | 31 (37.4) | 19 (22.9) | 5 (6.0) | 7 (8.4) |  |
| Comm Confidence (Routine) | 0 (0.0) | 4 (4.8) | 20 (24.1) | 45 (54.2) | 5 (6.0) | 9 (10.8) |  |

^a^ Factors are listed in descending order by the percentage of nurses that selected “Strongly Agree”.

^b^ This item was reverse coded, meaning if nurses disagreed that politicization hindered SAVEs, we treated this as an indication that nurses agreed that politicization did not affect their ability to hold SAVEs.

**Table 4A.** Barriers influencing nurses’ ability to hold SAVEs (n=179)

| **Barrier**^a^ | **Could not assess** | **Not a reason** | **Somewhat important** | **Very important** | **Select illustrative context for potential barriers to holding SAVEs, paraphrased from free-text responses** |
| --- | --- | --- | --- | --- | --- |
|  | **n (%)** | | | |  |
| Access to Guidance | 28 (15.6) | 43 (24.0) | 43 (24.0) | 65 (36.3) | - One nurse “did not know [SAVEs were] an option or available.” - Another nurse did not know “anything about the process” or that they would be “allowed to offer” free vaccine clinics at schools. - “Lack of policies/guidelines” around holding an “event like this” was a barrier. |
| Leadership Support (Routine) | 33 (18.4) | 53 (29.6) | 34 (19.0) | 59 (33.0) |  |
| External Partnerships | 28 (15.6) | 57 (31.8) | 43 (24.0) | 51 (28.5) | - Schools that do not have a school-based health center lack an “established relationship with local medical providers or other agencies” who could offer school-based vaccination services. - Community partners “were going through a staffing crisis” which limited their capacity to assist. |
| Leadership Support (COVID) | 47 (26.3) | 60 (33.5) | 22 (12.3) | 50 (27.9) |  |
| Politicization | 39 (21.8) | 58 (32.4) | 37 (20.7) | 45 (25.1) | - One nurse noted that they considered the “political views” of their school district and opted not to hold SAVEs so they would not be perceived as getting “involved in how parents raise their children.” - The potential “liability or political backlash of offering vaccinations at schools” dissuaded nurses from holding SAVEs. |
| Staff Support (Routine) | 45 (25.1) | 66 (36.9) | 31 (17.3) | 37 (20.7) |  |
| Staff Time | 32 (17.9) | 51 (28.5) | 60 (33.5) | 36 (20.1) | - Nurses described “not enough support in schools to accomplish this.” One called it “a daunting task to set up” and noted that school staff lacked the time to help them hold SAVEs. - Schools are “very strapped on resources” and “do not have extra people to put toward phone calls and gathering paperwork.” |
| Staff Support (COVID)^b^ | 52 (29.2) | 68 (38.2) | 25 (14.0) | 33 (18.5) |  |
| Funds to Cover Costs | 57 (31.8) | 61 (34.1) | 28 (15.6) | 33 (18.4) |  |
| Nurse Time | 25 (14.0) | 82 (45.8) | 43 (24.0) | 29 (16.2) | - While one nurse noted that they were supportive of holding SAVEs, they “have so much on [their] plate. [They] do not want to organize it or run it.” - “Attempting to do this at one school would be a big time commitment” for a nurse who noted that they cover five or more schools at a time. - Nurses are “spread thin” and working across several different schools. |
| Comm Confidence (COVID) | 55 (30.7) | 54 (30.2) | 41 (22.9) | 29 (16.2) | - Low trust “between parents and medical professionals who push the COVID vaccine” hinders SAVE implementation. |
| Comm Confidence (Routine) | 39 (21.8) | 65 (36.3) | 49 (27.4) | 26 (14.5) | - There were “strong opinions on vaccines after COVID” among parents. - There was a notable shift in parents’ willingness to “fully immunize their students” after COVID, whereas “prior to COVID I never had problems.” |
| Parent Trust | 50 (27.9) | 69 (38.6) | 34 (19.0) | 26 (14.5) | - Lack of trust in vaccines and the government. - Some community members were noted to “not like to associate school with healthcare and feel this is an overstep.” |
| Insurance | 64 (35.8) | 73 (40.8) | 17 (9.5) | 25 (14.0) | - Checking whether students qualify for VFC vaccines [through the Vaccines for Children Program] “is a big consideration.” |
| School Space | 25 (14.0) | 98 (54.8) | 41 (22.9) | 15 (8.4) | - Particularly for schools located in dense urban areas, nurses felt it would be “difficult to have the space to provide a mobile vaccine clinic.” |
| Translation Capabilities | 26 (14.5) | 107 (59.8) | 37 (20.7) | 9 (5.0) | - Nurses noted challenges with obtaining parental permission, particularly if families spoke dozens of different languages. One nurse noted that their district worked hard to translate district websites and forms into the ten most commonly-spoken languages. |

^a^ Barriers are listed in descending order by the percentage of nurses that selected “Very Important.”

^b^ One nurse did not answer this question, resulting in n=178 for this barrier.
